# Predicting mechanical neck pain intensity in computer professionals using machine learning: identification and correlation of key features

**DOI:** 10.3389/fpubh.2024.1307592

**Published:** 2024-03-21

**Authors:** Fatima Khanum, Abdur Raheem Khan, Ashfaque Khan, Aafreen Aafreen, Akhlaque Ahmad Khan, Ausaf Ahmad, Syed Mohammad Fauzan Akhtar, Omar Farooq, Mohammad Abu Shaphe, Mohammed M. Alshehri, Fazal Imam Shahi, Abdulfattah S. Alqahtani, Ashwag Albakri, Sakher M. Obaidat

**Affiliations:** ^1^Department of Physiotherapy, Integral University, Lucknow, India; ^2^Department of Electrical Engineering, Integral University, Lucknow, India; ^3^Department of Community Medicine, IIMS&R, Integral University, Lucknow, India; ^4^IIMSR, IIAHSR, IIANSR, Integral University, Lucknow, India; ^5^Department of Electronics Engineering, Aligarh Muslim University, Aligarh, India; ^6^Department of Physical Therapy, College of Applied Medical Sciences, Jazan University, Jazan, Saudi Arabia; ^7^Deanship of E-Learning & Information Technology, Jazan University, Jazan, Saudi Arabia; ^8^Department of Health Rehabilitation Sciences, College of Applied Medical Sciences, King Saud University, Riyadh, Saudi Arabia; ^9^Department of Computer Science, College of Computer Science & Information Technology, Jazan University, Jazan, Saudi Arabia; ^10^Department of Physical Therapy and Occupational Therapy, Faculty of Applied Medical Sciences, The Hashemite University, Zaraq, Jordan

**Keywords:** anthropometry, cervical range of motion, computer professionals, machine learning, neck disability index, neck pain

## Abstract

**Introduction:**

Mechanical neck pain has become prevalent among computer professionals possibly because of prolonged computer use. This study aimed to investigate the relationship between neck pain intensity, anthropometric metrics, cervical range of motion, and related disabilities using advanced machine learning techniques.

**Method:**

This study involved 75 computer professionals, comprising 27 men and 48 women, aged between 25 and 44 years, all of whom reported neck pain following extended computer sessions. The study utilized various tools, including the visual analog scale (VAS) for pain measurement, anthropometric tools for body metrics, a Universal Goniometer for cervical ROM, and the Neck Disability Index (NDI). For data analysis, the study employed SPSS (v16.0) for basic statistics and a suite of machine-learning algorithms to discern feature importance. The capability of the kNN algorithm is evaluated using its confusion matrix.

**Results:**

The “NDI Score (%)” consistently emerged as the most significant feature across various algorithms, while metrics like age and computer usage hours varied in their rankings. Anthropometric results, such as BMI and body circumference, did not maintain consistent ranks across algorithms. The confusion matrix notably demonstrated its classification process for different VAS scores (mild, moderate, and severe). The findings indicated that 56% of the pain intensity, as measured by the VAS, could be accurately predicted by the dataset.

**Discussion:**

Machine learning clarifies the system dynamics of neck pain among computer professionals and highlights the need for different algorithms to gain a comprehensive understanding. Such insights pave the way for creating tailored ergonomic solutions and health campaigns for this population.

## 1 Introduction

Neck pain is a prevalent medical condition affecting a vast number of individuals worldwide. Although it may appear to be a minor issue at first glance, it can significantly impede daily activities and even progress into a debilitating physical disability. Our modern era, defined by technological advancements and digitization, sees many people, students, professionals, and others like—spending countless hours hunched over computers (1, 2). This increased computer usage, whether for learning, work, or leisure, has become a prime mechanism for escalating neck discomfort cases.

Despite extensive studies on neck pain in computer professionals, there remains a lack of comprehensive understanding of the interaction between physical factors, such as posture, and health conditions, such as obesity, and their collective impact on neck pain severity. Current research predominantly relies on subjective methods, which can be subjective or limited. Our study aimed to fill this gap by leveraging machine learning techniques to objectively analyze and correlate these factors, providing a more nuanced understanding of neck pain in computer professionals.

Our research objective was to identify the key features contributing to mechanical neck pain intensity in computer professionals using machine-learning techniques. These features are then related to gain insights into their interplay and impact. The expected outcomes include a refined model for predicting neck pain intensity, contributing to more effective prevention and management strategies for this demographic.

To set the stage for our methodology, we detail how machine learning was employed in our research. This includes the data collection process, specific machine learning techniques, and analytical approaches to understand the relationships between various factors contributing to neck pain. This systematic approach is critical for achieving our research objectives and is elaborated upon in the following section on the materials and methods.

However, why does this matter? Persistent neck issues can occur rapidly beyond obvious physical inconveniences, leading to many health outcomes. These range from limited mobility and muscular weaknesses to psychological associations such as emotional distress and overall reduced quality of life (3). Thus, understanding neck pain in the modern context involves addressing discomfort and ensuring overall well-being.

The posture is important in the debate on neck pain. Alignment of the spine and head, or the lack thereof, plays an important role in preventing or triggering neck pain (4, 5). Such an observation becomes startlingly evident in corporate and office settings, where employees, often tied to their desks for extended periods, account for nearly half of all neck pain incidents annually (6, 7). One frequent manifestation of this discomfort is a noticeable reduction in the cervical range of motion (ROM) (8, 9), which, in layperson’s terms, implies limited neck movement flexibility.

At present, the world is facing an increasing rate of overweight and obesity (10). Overweight and obesity are associated with various severe health complications such as heart disease, diabetes, and high blood pressure. An alarming rise in obesity, classified traditionally via Body Mass Index (BMI), is now considered through other measurements such as waist circumference (WC), waist-hip ratio (WHR), and neck circumference (11, 12). The increasing neck circumference in individuals, possibly influenced by sedentary lifestyles and prolonged sitting, may contribute to neck pain. Computer professionals require a more comprehensive evaluation of the impact of body measurements on musculoskeletal health. This necessity stems from the unique demands of their work, which often involve prolonged periods of sedentary behavior and computer use. It is crucial to understand how various body metrics, such as neck circumference and upper-body fat distribution, affect the musculoskeletal system. Such an approach will allow for the better identification and management of related health issues, including neck pain, which is prevalent in this occupational group (13, 14).

In parallel with these medical and health surveillance, data science, particularly machine learning, is experiencing a significant change (15). As our study delves into the nuances of neck pain within the demographics of computer professionals, we recognize the limitations inherent in traditional research strategies, often constrained by their dependence on self-reporting. Machine learning is a pivotal tool in this context. With its unique ability to recognize patterns, machine learning offers the ability of revealing hidden relationships within extensive data set relationships that conventional investigative methods might face.

The aim of this research was to use the potential of machine learning to dissect the multifaceted neck pain problem among computer professionals. We are not just looking at source pain triggers. Rather, the goal is to explain the complex interplay between contributing factors, offering a holistic understanding. Such an trial holds the promise of highlighting the problem and paving the way for targeted interventions and ergonomic adjustments. To significantly diminish the incidence and severity of neck pain and support a strong and more productive professional environment.

## 2 Materials and methods

### 2.1 Participants

This prospective cross-sectional study focused on individuals with complaints of neck pain and analyzed various variables related to Neck Pain Intensity. This design enables systematic data collection at a single time point and offers a comprehensive overview. One hundred younger adults were invited from the community using convenience sampling.

### 2.2 Eligibility criteria

The inclusion criteria for the study focused on individuals aged between 25 and 44 years, particularly computer professionals, who are more prone to neck-related issues due to prolonged computer use. To be included in the study, participants had to report mechanical neck pain and discomfort, especially if they had spent six or more hours on computer work daily over the past 2 years. This approach was used to identify individuals whose neck pain was likely due to professional activities.

To ensure a homogeneous sample focused on neck pain resulting from computer work, individuals with a history of neck surgery, cervical tumors, neuropsychological disorders, or radiculopathies were excluded from the study. Additionally, those who were unable or unwilling to provide signed informed consent were excluded.

### 2.3 Study design

This prospective cross-sectional study was conducted within the computer center setting and in various departments of Integral University. The ethical committee of the IIMSR, Integral University, Lucknow, India, approved this study (approval number: IEC/IIMS&R/2022/65), ensuring compliance with the Helsinki Declaration. All participants involved in the study completed and signed an informed consent form in accordance with the ethical guidelines. The study spanned July 2022 to August 2023, comprehensively examining the variables of computer professionals aged between 25 and 44 years.

### 2.4 Sample size calculation

The sample size was determined using the following formula: *n* = z^2 *p* (1−*p*)/d^2, where Sample size = *n*, *p* = 50.0%, confidence level 95%, so Z score = 1.96 Margin of error (d) = 10%, non-response = 10%, and the calculated sample size was 100. After careful consideration and adherence to ethical guidelines, we excluded 15 patients who met the specific exclusion criteria. Ten patients were excluded from the final sample because they were unwilling to provide signed informed consent. The final participant group consisted of 75 individuals, including 27 men and 48 women aged between 25 and 44 years.

### 2.5 All participants were subjected to measurement

#### 2.5.1 Visual analog scale measurement

The degree of pain was measured using the visual analog scale (VAS) (0–10).Participants were asked to rate their pain levels over the previous 7 days and record that number, found to be ≤3.4 cm for mild pain, 3.5–7.4 for moderate pain, and ≥ 7.5 for severe pain; previous study shows that visual analog scale (VAS) is a reliable and valid scale for the measurement of cervical pain (16).

#### 2.5.2 Anthropometric measurement

The participants’ weight (kg) and standing height (cm) were measured to the closest 0.1 kg and 0.1 cm, respectively, while wearing light clothing and without shoes. Body Mass Index was estimated using the formula: kg/m^2^ (weight in kilograms divided by squared height in meters). According to the Asia-Pacific cut-off criteria, the BMI was divided into four groups: underweight (18.5 kg/m^2^), normal weight (18.5–22.9 kg/m^2^), overweight (23–24.9 kg/m^2^), and obese (25 kg/m^2^). This categorization aligns with the findings of Romero-Corral et al. (17), who demonstrated the accuracy of BMI as a good outcome measure.

The circumference of the neck was measured using a rigid plastic measuring tape. The participants were measured while standing erect, gazing directly ahead, and keeping their shoulders down but not slouch. The measurement was made at a point halfway between the mid-cervical spine and mid-anterior neck, slightly below the level of the laryngeal prominence (Adam’s apple) to the nearest millimeter. This method is supported by the study of Anothaisintawee et al. (18), which indicates that neck circumference is a good anthropometric tool.

Waist and hip circumferences were measured to the nearest 0.1 cm using a flexible narrow no-stretch tape in adults wearing minimal clothing, standing straight but not pulling in their stomachs. Waist circumference was measured halfway between the lower ribs and iliac crest, while hip circumference was measured at the largest circumference around the buttocks, both of which are considered reliable and valid tools for assessing body composition (19).

#### 2.5.3 Cervical range of motion measurement

Universal Goniometer, a tool used most commonly for evaluating joint ROM in the clinical settings, comes out as a simple alternative for global use at low price. Notably, the reliability and validity of cervical goniometric measurements are considered excellent, ensuring accurate measurement of cervical range of motion (20).

The following movements were recorded: sagittal flexion/extension, lateral flexion, and lateral rotation (All six movements were recorded). In sagittal flexion and extension, the participants were required to make a “double chin” (sub occipital flexion), flex fully, nodding the head back, and then fully extending. In lateral flexion, the subjects fixed their gaze on a set point directly ahead and were observed while laterally flexing both to the right and left. In lateral flexion, the subject looked to the right and then to the left while holding a horizontal gazes parallel to the floor. Prior to the measurement process, a demonstration of the movements to be studied was provided prior to the definitive measurement and then repeated by the subject. This facilitated the examination and allowed the neck muscles and ligaments to “warm up.” Maximal movement was recorded as that achieved at the onset of tightness, discomfort, or secondary movement, for example, shoulder tilt or head rotation, when assessing lateral flexion.

#### 2.5.4 Neck disability index

The Neck Disability Index (NDI) is a ten-item questionnaire that measures the disability caused by whiplash and neck pain and is based on the Oswestry Low Back Pain Index. There are six measures pertaining to activities of daily life: lifting, working, driving, having fun, taking care of one person, and reading. Four measures were related to subjective symptomatology: pain intensity, headache, concentration, and sleep. The administration of the questionnaire requires no additional training and only takes 5–10 min to complete and score. It is important to note that previous research has demonstrated good reliability and validity of the NDI, making it a reliable tool for assessing disability related to neck pain (21).

Participant Instructions and Scoring: Each question had six possible responses, ranging from no disability (zero) to entire impairment (six) (5). The overall score ranges from 0 (no disability) to 50, when the 10 components are added together (maximum disability). This rating was expressed as a percentage. A score of less than 4 denotes no disability, a score of 5–14 indicates light disability, a score of 15–24 indicates moderate disability, a score of 25–34 indicates severe disability, and a score of more than 35 denotes complete disability.

### 2.6 Statistical analysis

Analyzes were performed using SPSS (v16.0). Descriptive statistics were used to determine participants’ VAS scores. Variance in baseline traits by VAS categories was assessed via ANOVA and chi-square, with *p* < 0.05. considered significant. Feature significance was evaluated using five machine learning algorithms: MRMR, Chi2, Relief, ANOVA, and Kruskal Wallis, providing comparative feature rankings. MRMR Algorithm (Minimum Redundancy Maximum Relevance) MRMR aims to select features that are both highly relevant to the target variable and have minimal redundancy. It utilizes mutual information (MI) and redundancy measures for feature selection.Chi2 Algorithm (Chi-Square) Chi2 assesses the independence between categorical features and the target variable by comparing observed and expected frequencies in a contingency table.

ReliefF Algorithm ReliefF assesses the feature importance by comparing the feature values of the nearest neighbors for instances of different classes. For a dataset with instances belonging to different classes, ReliefF updates the feature weights based on the difference in feature values between the instances and their nearest neighbors. ANOVA Algorithm (Analysis of Variance) ANOVA assesses the significance of mean differences among groups, identifying features with varying means important for classification. The ANOVA F-statistic was calculated as the ratio of the between-group variance to the within-group variance. The Kruskal-Wallis test is a non-parametric test assessing differences in medians among groups, suitable for non-normally distributed data. For a dataset with multiple groups, Kruskal-Wallis tests whether the medians of these groups are significantly different based on the ranks of the observations.

**Figure 1:**
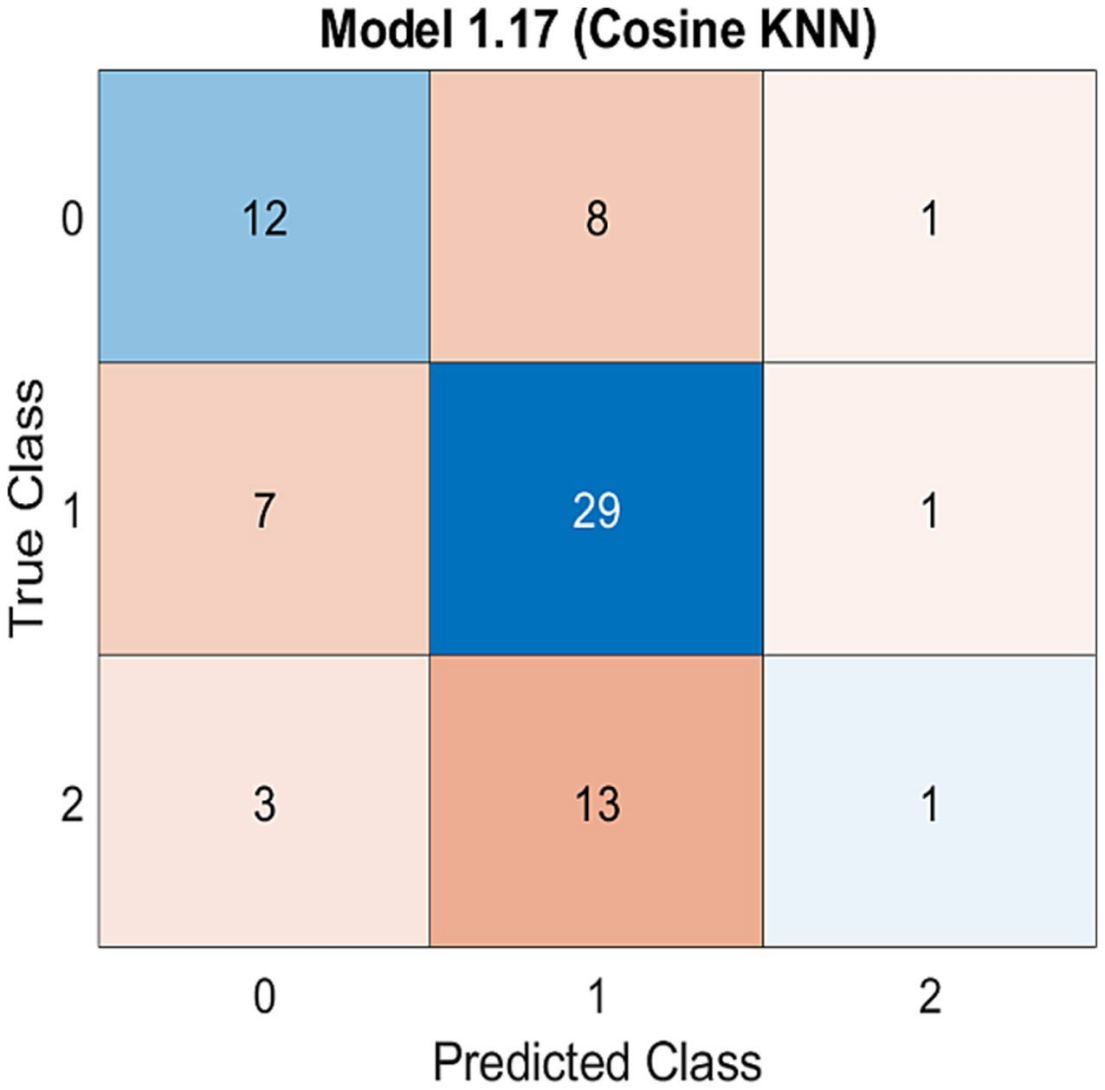
Confusion matrix model for three classes of VAS score (mild, moderate and severe) using KNN machine learning algorithm.

Step-by-step methods were used to conduct and interpret the confusion matrix for evaluating the KNN algorithm’s performance in classifying VAS scores into three classes: mild, moderate, and severe. These are as follows:

Step 1: Data preparation: ensure that you have a labeled test dataset containing VAS scores and the corresponding true severity classes (mild, moderate, and severe).
Step 2: Implement the KNN algorithm: apply the KNN algorithm to the test dataset to predict the severity classes based on the VAS scores.
Step 3: Create the confusion matrix: create a confusion matrix, a square matrix that displays the model’s performance by comparing the predicted classes against the true classes.
Step 4: Populate the confusion matrix: count the number of observations that fall into each combination of true and predicted severity classes. The confusion matrix was populated by these counts. The diagonal elements represent correct predictions (true positives) and the off-diagonal elements represent misclassifications (false positives or false negatives).
Step 5: visualize the confusion matrix (Figure 1): create a visual representation of the confusion matrix, highlighting the arrangement of true and predicted severity classes.

## 3 Results

Table 1 summarizes the characteristics of our study participants stratified by age, gender, and severity of neck pain due to computer use. The data show a diverse range of participants, mostly within the 31–40 age groups, suggesting this group’s significant representation in our study. Notably, the distribution across the mild, moderate, and severe neck pain categories indicated a higher prevalence of moderate neck pain among our participants. Gender analysis showed a greater number of females in the moderate neck pain category.

**Table 1:** Distribution of participant characteristic.

Criteria	Group	Mild (*n* = 22)	Moderate (*n* = 51)	Severe (*n* = 2)	Total (*n* = 75)	*p* value
Age group	25–30	4	18	0	22	0.34
	31–40	14	27	1	42	
	41–50	4	6	1	11	
Gender	Male	4	22	1	27	0.11
	Female	18	29	1	48	
Computer Work	Mean ± SD	7.2 ± 1.4	7.8 ± 2.4	7.0 ± 0.0	7.6 ± 2.2	0.61

Table 2 presents the results of the feature significance evaluation using five distinct machine learning algorithms: MRMR, Chi2, ReliefF, ANOVA, and Kruskal Wallis. Each algorithm generates rankings for the features based on their importance in the context of the study. For the age category, MRMR ranked age as 0, indicating its significance in relation to the target variable. Chi2 and ReliefF assigned lower ranks, whereas ANOVA and Kruskal Wallis ranked Age as 1, suggesting consistency in recognizing its importance across algorithms. For BMI, MRMR, ANOVA, and Kruskal Wallis assigned a rank of 0 to BMI, indicating high significance. Chi2 and ReliefF were assigned lower ranks, but the differences in ranks were minimal. ReliefF and ANOVA ranked the number of hours spent on computer work as the most significant feature (rank 12), while MRMR, Chi2, and Kruskal Wallis provided varying ranks but still acknowledged its importance. All five algorithms consistently ranked the NDI Score as the most significant feature, with a rank of 1. The high ranks and corresponding values indicate their crucial role in classification. ReliefF and ANOVA recognized neck circumference as highly significant (rank 7), while MRMR, Chi2, and Kruskal Wallis provided varying ranks. Waist and hip circumferences received mixed rankings across algorithms, with some assigning lower importance (rank 13). Most algorithms assigned intermediate ranks to these cervical ranges of motion (Forward Flexion, Extension, Right/Left Lateral Flexion, Right/Left Rotation) features, and Chi2 consistently recognized the importance of lateral flexion and rotation. Among all, the NDI Score as the most significant feature provides confidence in its relevance, whereas variations in rankings for other features underscore the nuanced perspectives offered by different algorithms.

**Table 2:** Interpretation of ranking classification using five different machine learning algorithms.

S. No.	Features	MRMR Algorithm	Chi2 Algorithm	ReliefF Algorithm	Anova Algorithm	Kruskalwallis Algorithm
1	Age	0	Rank # 11 (0.2495)	Rank # 13 (−0.0198)	Rank # 8 (1.0210)	Rank # 7 (1.0273)
2	BMI	0.0000	Rank # 12 (0.1936)	Rank # 4 (0.0042)	Rank # 5 (1.7199)	Rank # 6 (1.6312)
3	No. of Hours (Comp work)	Rank # 4 (0.0037)	Rank # 3 (1.7440)	Rank # 12 (−0.0099)	Rank # 7 (1.5626)	Rank # 5 (2.0898)
4	NDI Score (%)	Rank # 1 (0.0948)	Rank # 1 (4.6458)	Rank # 1 (0.0347)	Rank # 1 (12.9037)	Rank # 1 (12.8256)
5	Neck circumference (cm)	Rank # 2 (0.0283)	Rank # 9 (0.3302)	Rank # 7 (−0.0022)	Rank # 3 (1.9030)	Rank # 4 (2.2225)
6	Waist circumference (cm)	0	Rank # 13 (0.0794)	Rank # 8 (−0.0032)	Rank # 9 (0.9104)	Rank # 9 (0.7987)
7	Hip circumference (cm)	0	Rank # 7 (0.3827)	Rank # 6 (−0.0013)	Rank # 13 (0.0831)	Rank # 13 (0.3095)
8	C. forward flexion	0	Rank # 10 (0.2777)	Rank # 2 (0.0174)	Rank # 10 (0.5934)	Rank # 10 (0.7183)
9	C. extension	0	Rank # 5 (0.6335)	Rank # 9 (−0.0053)	Rank # 11 (0.4406)	Rank # 11 (0.5000)
10	Right lateral flexion	Rank # 5 (0.0028)	Rank # 2 (1.7532)	Rank # 11 (−0.0086)	Rank # 4 (1.8748)	Rank # 3 (2.4680)
11	Left lateral flexion	0	Rank # 8 (0.3567)	Rank # 10 (−0.0081)	Rank # 12 (0.3520)	Rank # 12 (0.3472)
12	Right Rotation	Rank # 6 (0.0005)	Rank # 6 (0.5345)	Rank # 5 (0.0034)	Rank # 2 (3.8389)	Rank # 2 (4.1240)
13	Left Rotation	Rank # 3 (0.0126)	Rank # 4 (1.7354)	Rank # 3 (0.0081)	Rank # 6 (1.5633)	Rank # 8 (0.8585)

The capability of the KNN algorithm to classify VAS scores was gaged using a confusion matrix, highlighting the accuracy of the model (Figure 1). The confusion matrix is used to evaluate the efficacy of classification models with respect to a specific test dataset. The determination of true values for test data is a prerequisite for accurate determination. KNN algorithm was employed to assess its ability to classify VAS scores into three classes: mild, moderate, and severe. This classification was performed using a confusion matrix, which is a performance evaluation tool that allows for a detailed analysis of the model’s predictions across different classes. By employing the KNN algorithm and visualizing the confusion matrix, we evaluated the accuracy of the model for classifying different levels of neck pain severity. The confusion matrix provides insights into how well the model predicts the true severity class for each observation. Where the confusion matrix is likely to be visualized, the arrangement of true classes and predicted classes allows for an assessment of the model’s performance. The diagonal elements of the matrix represent correct predictions (true positives), whereas the off-diagonal elements represent misclassification (false positives or false negatives). The distribution of these elements helps to quantify the accuracy, precision, recall, and other performance metrics of the model. It directly addressed the relationship between the predicted and actual severity of neck pain. This provides a clear understanding of how well the model captures the nuances in the data and whether it can effectively distinguish between mild, moderate, and severe neck pain based on the influencing factors under investigation.

**Figure 2:**
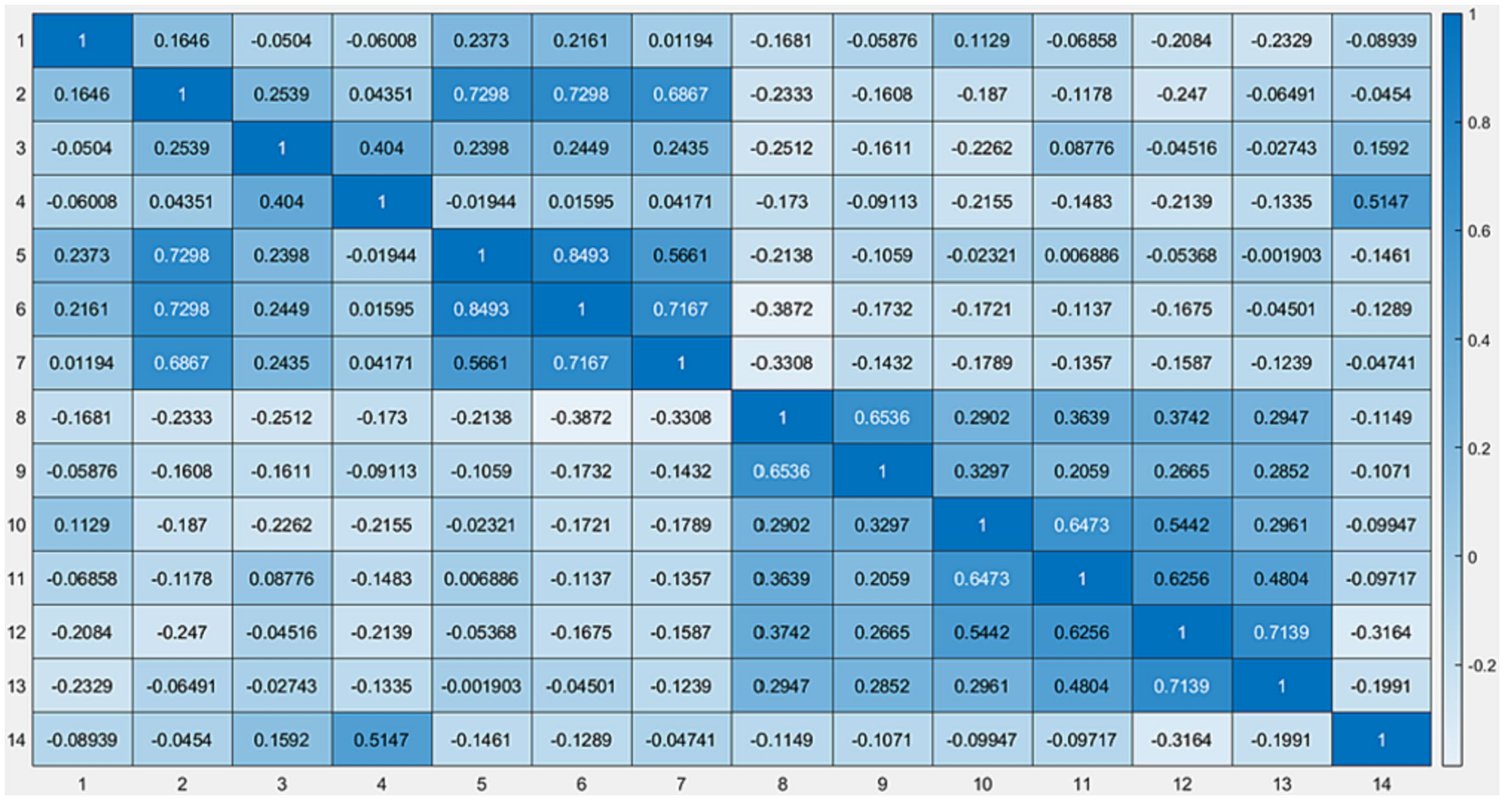
Heat map data representation using machine learning.

Figure 2 presents a visual summary of the correlation of the variables of the data used in this study in the form of a heat map, which visually displays the relationships between the study variables mentioned in Table 2, numbered 1 to 13. This visual representation is in direct alignment with the predominant objective of the study, which is to investigate the relationship between neck pain intensity, anthropometric metrics, cervical range of motion, and related disability. The heatmap provides a comprehensive overview of how these variables are interconnected and the degree to which they influence one another. In addition, we offer an at-a-glance depiction of correlation strength and direction. Strong positive correlations denoted by high positive “r” values, conversely, strong negative correlations, represented by high negative “r” values, values close to zero indicate a weak or negligible correlation. By specifically incorporating a heat map for correlations, this study emphasizes a nuanced exploration of relationships, highlighting the intricate connections between neck pain intensity, anthropometric metrics, cervical range of motion, and related disability. The heat map identifies potential patterns, dependencies, or areas of interest within the dataset. A strong positive correlation between neck pain intensity and related disabilities might underscore the impact of pain on functional limitations. Conversely, a lack of correlation between certain anthropometric metrics and cervical range of motion suggests that these variables are relatively independent of each other. By incorporating correlation coefficients, the study explored the intricate relationships between neck pain intensity, anthropometric metrics, cervical range of motion, and related disability.

## 4 Discussion

In the ranking classification generated by five distinct machine learning algorithms, several features showed varying rankings across the algorithms. The details of the ranking classifications are summarized in Table 2. The “NDI Score (%)” consistently emerged as the top-ranked feature across Chi2, ReliefF, Anova, and Kruskal Wallis algorithms, high-lighting its primary significance in the dataset. This consistently high ranking across multiple algorithms underscores the importance of NDI Scores in assessing neck pain severity.

The ‘Age’ feature displayed varying rankings, which were not consistent across all algorithms. It was not ranked by MRMR, while it received lower rankings by Chi2 and ReliefF and somewhat higher rankings by ANOVA and Kruskal Wallis. This variation in the ranking of ‘Age’ across algorithms suggests that its impact on neck pain severity is more complex and may be influenced by interactions with other variables in the dataset.

The number of computer work hours was ranked significantly by Chi2 and MRMR but not by ReliefF. These variations point to the different methodologies used by each algorithm to assess feature importance and indicate that the relationship between work hours and neck pain is multifaceted.

Regarding anthropometric measures, ‘BMI’ was not ranked by MRMR but was considered moderately significant by other algorithms. Similarly, neck, waist, and hip circumferences had inconsistent rankings, indicating that their impact on neck pain might vary depending on the specific algorithm used, reflecting their data-dependent or algorithm-specific significance.

The disparities in feature rankings highlight the importance of leveraging multiple algorithms in machine learning to gain a more comprehensive understanding of feature importance in complex datasets like ours.

The confusion matrix (Figure 1) is a visual representation that provides insight into the accuracy and precision of predictions by comparing the actual classes to those predicted by the model.

The utilization of a confusion matrix enables the computation of various model parameters, including, but not limited to, accuracy and precision. The findings of the present investigation indicate that the validation prediction of the Visual Analog Scale (VAS) score across three categories, namely mild, moderate, and severe, was 56%. This 56% prediction accuracy implies that over half of the pain intensity cases, as measured by the VAS, can be accurately predicted by the dataset. In a clinical or practical setting, this level of accuracy suggests that the KNN algorithm, while moderately effective, may require further optimization for more reliable use in predicting neck pain severity. The existence of a significant class imbalance in the data, likely due to the limited sample size, also impacts the predictive accuracy of the models.

The rows of the matrix typically represent the actual classes, whereas the columns represent the predicted classes. The diagonal elements of the matrix running from the top left to the bottom right depict the number of correct predictions for each class. Ideally, for a perfect model, these diagonal values would be the highest in their respective rows, indicating ac-curate predictions, while all other values would be zero, signifying no misclassifications.

In contrast, off-diagonal elements represent instances in which the model predictions differ from the actual values. For instance, a value in the cell at the intersection of the ‘Actual: Mild’ row and ‘Predicted: Moderate’ column would indicate the number of instances where the model inaccurately classified a ‘Mild’ score as ‘Moderate.’

By examining the distribution and magnitude of the values in the confusion matrix, we can discern the efficacy of the KNN models in predicting the VAS scores. Higher numbers on the matrix diagonal suggest successful predictions, whereas significant numbers outside the diagonal highlight the potential areas of misclassification. This observation is crucial for clinicians and practitioners who might utilize this model, as it indicates the need for cautious interpretation of the results, especially in cases where algorithm prediction does not align with clinical assessments. Therefore, while the KNN algorithm provides a useful tool for preliminary assessment, it should be employed in conjunction with clinical judgment and other diagnostic methods for comprehensive evaluation of neck pain severity.

Figure 2 presents a visual summary of the correlations between the variables of the data used in this study. The correlation coefficient is denoted by “r” presents a heat map, which offers a graphical representation of data where individual values are depicted using color gradients. This visualization technique is particularly effective for understanding the patterns, variances, and anomalies in large datasets. In the context of machine learning, heat maps can be instrumental in highlighting the feature importance, correlations between variables, or even understanding the distribution and concentration of data points in a multidimensional space.

The colors in the heat map usually range from cool to warm (e.g., blue to red or green to red) to signify low and high values, respectively. By examining color intensity and distribution across the map, readers can quickly discern patterns and relationships.

For instance, in the case of representing feature importance, areas or cells of the heat map that glow with warmer colors might denote features that have a more significant influence on the decision-making process of a machine learning model. However, cooler regions may suggest less impactful or irrelevant features.

If the heat map illustrates correlations, warmer colors might represent strong positive correlations between variables, whereas cooler colors could denote negative correlations. A neutral color, often white or gray, indicated little to no correlation.

A heat map visually summarizes the correlations between variables in our study (Figure 2). This heat map offers a powerful tool for identifying patterns and relationships in the data, with color intensity indicating the strength of the correlations. This visualization helps in understanding the complex interplay between neck pain intensity, anthropometric metrics, cervical range of motion, and related disabilities, offering insights that might be less apparent in numerical data.

Our primary objective in this study was to decipher the intricate relationships between neck pain severity and several potential influencing factors among computer professionals. We used the visual analog scale (VAS) as our main evaluative tool. Through advanced machine learning techniques, our goal was to define these associations, emphasizing our hypothesis that prolonged screen exposure and specific anthropometric measurements significantly affect neck pain severity in our target population.

A striking result supporting our hypothesis was that an overwhelming 69% of computer professionals reported moderate neck pain (22). The intensive use of computers has complications in the musculoskeletal system. Especially when considering prolonged screen exposure accentuated by poor ergonomics and infrequent breaks, our findings concur with prior studies underscoring the aggravation of musculoskeletal symptoms under similar conditions (23).

Our detailed investigation into anthropometric metrics such as BMI and neck, waist, and hip circumferences showed that their significance varied across different machine learning algorithms. Some algorithms, for example, might stress the influence of BMI in predicting neck pain, whereas others may subordinate it when juxtaposed with other determinants (24). This diversity underlines the pivotal role of algorithmic diversity in deriving outcomes, and parallels findings from other studies that emphasize the multifaceted nature of machine learning in healthcare predictions.

Notably, the Neck Disability Index (NDI) score was consistent across most algorithms. Echoing earlier research, the NDI, representing neck-related disabilities, captures a myriad of underlying factors that contribute to neck pain (25).

While our primary focus was on screen exposure and anthropometric measures, our algorithms also identified age as a notable factor influencing neck pain. Although age might sometimes be eclipsed by more immediate determinants such as posture or screen time, it is an integral component of the overarching assessment framework, a sentiment shared by Jenkins et al. (26) in their exploration of age-related neck pain among computer users.

This study has some limitations. This study is limited by its reliance on a single cross-sectional design, which, while offering valuable immediate insights, needs to establish causality or track temporal changes, necessitating longitudinal studies for a deeper understanding. The sample size of 75 participants, selected through convenience sampling, may not fully represent the broader population of computer professionals, potentially introducing a selection bias. The subjective nature of self-reported measures, such as the visual analog scale (VAS), could also affect the accuracy of the findings. Additionally, despite their analytical power, the use of machine learning algorithms carries challenges such as complexity in interpretation and moderate predictive accuracy, underscoring the need for further optimization and caution in their application. The study focus on a specific demographic and limited exploration of external factors like ergonomics, environmental and physical activity further restricts the generalizability of its results, highlighting the need for a more comprehensive approach in future research.

The application of machine learning in our research highlights the importance of employing an array of algorithms for a holistic overview. The confusion matrix, for instance, highlighted both the efficacy and areas requiring refinement in the VAS score classification, underscoring the precision of machine learning applications (26). Our models also suggest an intriguing correlation between neck circumference and upper body fat distribution, a subject that requires further examination in broader metabolic health discussions (27, 28). In brief, while resonating with the findings of previous studies, our research underscores the interplay between multiple factors influencing neck pain among computer professionals. As our professional landscape grows increasingly digital, insights such as these become instrumental in creating both preventive and therapeutic interventions.

To advance the understanding and management of neck pain among computer professionals, future research should consider longitudinal studies to explore the temporal relationships and causality among computer use, anthropometric factors, and neck pain. Investigating the efficacy of specific ergonomic interventions and workplace adjustments in real-world regions could provide actionable insights for reducing neck pain in this population. Additionally, investigating the role of personalized medicine, such as tailored physical therapy programs and ergonomic recommendations based on individual risk factors (e.g., BMI, neck circumference, and computer usage patterns), could enhance the accuracy of prevention and treatment strategies. The inclusion of machine learning models, such as those used in our study, could assist clinicians in identifying patients at a higher risk of developing neck pain, thereby enabling early intervention.

## 5 Conclusion

Our study, “Predicting Mechanical Neck Pain Intensity in Computer Professionals using Machine Learning,” illustrates the critical factors contributing to neck pain among computer professionals. We found a significant impact of the Neck Disability Index (NDI) score, the influence of variables such as age and computer work hours, and the importance of anthropometric measures, including BMI and neck circumference. These insights highlight the complexity of neck pain and demonstrate the role of machine learning in explaining intricate relationships in health data. For computer professionals, our findings emphasize the necessity of ergonomic awareness and implementation of specific preventative measures against neck pain. The factors identified in our study support a nuanced approach to the diagnosis and management of neck pain in healthcare settings. From a practical perspective, the outcomes of our research support the formation of ergonomic solutions and health initiatives specifically designed to mitigate the risks faced by computer professionals. Additionally, our results suggest that healthcare professionals should consider a broad spectrum of factors when evaluating and treating neck pain in this group.

## Data availability statement

The original contributions presented in the study are included in the article/supplementary material, further inquiries can be directed to the corresponding author.

## Ethics statement

The ethical committee of IIMSR, Integral University, Lucknow, India (approval number: IEC/IIMS&R/2022/65) approved this study. All experiments were carried out in line with the Helsinki Declaration. An informed consent form was completed and signed by all participants. This work is acknowledged under Integral University manuscript communication number IU/R&D/2023-MCN0002019.

## Author contributions

FK: Writing – original draft, Software, Resources, Formal analysis, Data curation. AbK: Writing – review & editing, Writing – original draft, Visualization, Validation, Supervision, Resources, Project administration, Methodology, Investigation, Formal analysis, Data curation, Conceptualization. AsK: Writing – review & editing, Supervision, Resources, Investigation, Formal analysis. AA: Writing – review & editing, Supervision, Project administration, Funding acquisition. AkK: Writing – review & editing, Visualization, Software, Resources, Investigation, Formal analysis, Data curation. AuA: Writing – review & editing, Methodology, Investigation. SA: Writing – review & editing, Resources, Methodology, Formal analysis, Data curation, Conceptualization. OF: Writing – review & editing, Validation, Software, Resources, Data curation, Conceptualization. MS: Writing – review & editing, Methodology, Formal analysis, Conceptualization. MA: Writing – review & editing, Methodology, Investigation, Formal analysis. AbA: Writing – review & editing, Investigation, Funding acquisition, Formal analysis, Data curation, Conceptualization. AsA: Writing – review & editing, Resources, Project administration, Methodology, Investigation. SO: Data curation, Formal analysis, Investigation, Visualization, Writing – review & editing. FS: Data curation, Writing – review & editing.
